# Baltimore College of Dental Surgery

**Published:** 1840

**Authors:** 


### BALTIMORE COLLEGE OF DENTAL SURGERY.

We have received the "*First Annual Announcement*," of the Board of Visitors of an institution under the above title, in the city of Baltimore. The venerable Dr. Thos. E. Bond, is at the head of the Board, which embraces many respectable gentlemen, both in and out of the profession.

The faculty consists of,

*Horace H. Hayden*, M. D., Prof. of Dental physiology and pathology.

*H. Willis Baxley*, M. D., Prof. of Anatomy and physiology.

*Chappin A. Harris*, M. D., Prof. of Practical Dentistry.

*Thos. E. Bond, Jr.*, M. D., Prof. of Special Pathology and theraputics.

We should have greater faith in the success of this very laudable undertaking, if the number engaged in it were less. A charter has been obtained, and as far as we know, this is the first attempt to found a school of instruction for this particular branch of the profession, which has been made in the United States. That such a school is much needed, no one will deny, who knows that in most of our regular medical colleges, the diseases of the teeth are scarcely mentioned. The course of instruction will commence the 1st Monday of November, and continue four months. We observe that a charter has been obtained, and that the visitors are authorized to confer the degree of Doctor of Dental Surgery. This novel enterprize has our best wishes, and we shall be happy to contribute all that may be in our power to its permanent establishment. D.

